# Supplementary material for: Prioritizing countries for TB vaccine readiness research using a global stakeholder-centric approach
Source: PLOS Glob Public Health. 2025 Aug 1;5(8):e0004668. doi: 10.1371/journal.pgph.0004668 (PMC12316289; doi:10.1371/journal.pgph.0004668)
Supplement: S1 Table — (DOCX) [file pgph.0004668.s001.docx]

| **Country** | **Country Identifier Code** |
| --- | --- |
| **Bangladesh** | **18** |
| **Burma/Myanmar** | **5** |
| **Cambodia** | **3** |
| **DRC** | **19** |
| **Ethiopia** | **20** |
| **India** | **7** |
| **Indonesia** | **12** |
| **Kenya** | **16** |
| **Kyrgyzstan** | **21** |
| **Malawi** | **22** |
| **Mozambique** | **4** |
| **Nigeria** | **11** |
| **Pakistan** | **6** |
| **Philippines** | **9** |
| **South Africa** | **23** |
| **Tajikistan** | **13** |
| **Tanzania** | **2** |
| **Uganda** | **14** |
| **Ukraine** | **15** |
| **Uzbekistan** | **10** |
| **Vietnam** | **1** |
| **Zambia** | **17** |
| **Zimbabwe** | **8** |

**S1 Table. Key for anonymized country designations**
