# Supplementary material for: Prioritizing countries for TB vaccine readiness research using a global stakeholder-centric approach
Source: PLOS Glob Public Health. 2025 Aug 1;5(8):e0004668. doi: 10.1371/journal.pgph.0004668 (PMC12316289; doi:10.1371/journal.pgph.0004668)
Supplement: S2 Table — (DOCX) [file pgph.0004668.s002.docx]

**S2 Table. Stakeholder recruitment and response rate by country**

| **Country** | **Targeted (n)** | **Responded (n)** | **Response Rate (%)** |
| --- | --- | --- | --- |
| Bangladesh | 6 | 4 | 66.7 |
| Cambodia | 22 | 6 | 27.3 |
| DRC | 17 | 5 | 29.4 |
| Ethiopia | 14 | 6 | 42.9 |
| India | 37 | 6 | 16.2 |
| Indonesia | 15 | 5 | 33.3 |
| Kenya | 20 | 5 | 25.0 |
| Kyrgyzstan | 9 | 7 | 77.8 |
| Malawi | 17 | 5 | 29.4 |
| Mozambique | 12 | 4 | 33.3 |
| Myanmar | 1 | 0 | 0.0 |
| Nigeria | 15 | 9 | 60.0 |
| Pakistan | 8 | 1 | 12.5 |
| Philippines | 10 | 4 | 40.0 |
| South Africa | 26 | 4 | 15.4 |
| Tajikistan | 9 | 2 | 22.2 |
| Tanzania | 19 | 4 | 21.1 |
| Uganda | 30 | 6 | 20.0 |
| Ukraine | 5 | 2 | 40.0 |
| Uzbekistan | 7 | 2 | 28.6 |
| Vietnam | 9 | 2 | 22.2 |
| Zambia | 16 | 7 | 43.8 |
| Zimbabwe | 28 | 5 | 17.9 |
| Global, other countries | 75 | 14 | 18.7 |
| **Total** | **427** | **115** | **26.9** |
