# Supplementary material for: Prioritizing countries for TB vaccine readiness research using a global stakeholder-centric approach
Source: PLOS Glob Public Health. 2025 Aug 1;5(8):e0004668. doi: 10.1371/journal.pgph.0004668 (PMC12316289; doi:10.1371/journal.pgph.0004668)
Supplement: S3 Table — (DOCX) [file pgph.0004668.s003.docx]

**S3 Table. Mean criterion weights and corresponding 95% confidence intervals for the 17 prioritization criteria derived from stakeholder BWS responses and incorporating a direct anchoring approach.** The anchor, equal to zero, indicates the absolute importance of criteria, where positive weights above the anchor are considered an “important” criterion for country prioritization, while negative values below the anchor are considered an “unimportant” criterion for country prioritization.

| **Criteria** | **Mean criteria weight (95%CI) *** |
| --- | --- |
| **Overall TB burden** | 51.2 (47.0-55.5) |
| **Political commitment to end TB** | 46.7 (40.7-52.7) |
| **Burden of TB-related deaths** | 35.7 (30.3-41.1) |
| **Health systems strength** | 34.9 (30.3-39.5) |
| **Adult COVID-19 coverage** | 31.9 (27.1-36.6) |
| **Favorable regulatory processes** | 30.5 (26.0-35.0) |
| **TB burden among children** | 29.2 (24.6-33.8) |
| **Financial commitment to TB** | 28.0 (22.0-34.0) |
| **Burden of drug resistant-TB** | 27.8 (22.5-33.1) |
| **Infant DPT3 coverage** | 26.3 (22.0-30.7) |
| **Gavi eligibility** | 24.1 (18.4-29.9) |
| **Adolescent HPV vaccine introduction** | 20.3 (15.6-25.0) |
| **Availability of short-course TPT regimens** | 17.8 (11.8-23.8) |
| **Infant BCG coverage** | 14.8 (10.3-19.3) |
| **HIV-associated TB burden** | 14.0 (9.6-18.3) |
| **Participation in TB vaccine trials** | 9.2 (3.6-14.8) |
| **Importance threshold (i.e., anchor)** | 0 |
| **Vaccine manufacturing capacity** | -16.9 (-23.2, -10.5) |

DPT3: diphtheria-pertussis- tetanus; HPV: human papillomavirus; TPT: TB preventive therapy; BCG: Bacille Calmette-Guérin

*Weights are zero-anchored interval scaled.
