# Supplementary material for: Prioritizing countries for TB vaccine readiness research using a global stakeholder-centric approach
Source: PLOS Glob Public Health. 2025 Aug 1;5(8):e0004668. doi: 10.1371/journal.pgph.0004668 (PMC12316289; doi:10.1371/journal.pgph.0004668)
Supplement: S4 Table — (DOCX) [file pgph.0004668.s004.docx]

**S4 Table. Mean criterion weights and corresponding 95% confidence intervals for the 16 final prioritization criteria deemed important, derived from stakeholder BWS responses**

| **Criteria** | **Mean criterion weight**  **(95%CI)*** |
| --- | --- |
| **Overall TB burden** | 11.1 (10.2-12.0) |
| **Political commitment to end TB** | 10.3 (9.4-11.3) |
| **Burden of TB related deaths** | 7.9 (7.0-8.8) |
| **Health systems strength** | 7.5 (6.7-8.3) |
| **Adult COVID-19 vaccine coverage** | 7.4 (6.5-8.3) |
| **TB burden among children** | 6.6 (5.7-7.4) |
| **Burden of drug resistant-TB** | 6.3 (5.4-7.1) |
| **Favorable regulatory processes** | 6.2 (5.5-7.0) |
| **Financial commitment to TB** | 6.2 (5.4-7.0) |
| **Infant DPT3 coverage** | 5.8 (5.0-6.6) |
| **Gavi eligibility** | 5.1 (4.3-5.8) |
| **Short-course TPT introduction** | 4.6 (3.9-5.4) |
| **Adolescent HPV introduction** | 4.5 (3.9-5.1) |
| **HIV-associated TB burden** | 3.8 (3.1-4.5) |
| **Infant BCG coverage** | 3.6 (3.1-4.1) |
| **Participation in TB vaccine trials** | 3.2 (2.5-3.9) |

DPT3: diphtheria-pertussis- tetanus; TPT: TB preventive therapy; HPV: human papillomavirus; BCG: Bacille Calmette-Guérin

*Weights are probability rescaled and collectively sum to 100.
