# Supplementary material for: Prioritizing countries for TB vaccine readiness research using a global stakeholder-centric approach
Source: PLOS Glob Public Health. 2025 Aug 1;5(8):e0004668. doi: 10.1371/journal.pgph.0004668 (PMC12316289; doi:10.1371/journal.pgph.0004668)
Supplement: S5 Table — (DOCX) [file pgph.0004668.s005.docx]

**S5 Table. Mean criterion weights and corresponding 95% confidence intervals for the final 16 prioritization criteria derived from stakeholder BWS responses by geographic region**

| Statement | Africa  (n=60) | Asia  (n=42) | Other  (n=13) |
| --- | --- | --- | --- |
| Overall TB burden | 10.3  (9.0-11.5) | 12.1  (10.7-13.4) | 11.6  (8.8-14.5) |
| Political commitment to end TB | 9.9  (8.7-11.2) | 11.0  (9.5-12.4) | 10.0  (6.8-13.2) |
| Burden of TB-related deaths | 7.9  (6.5-9.1) | 7.6  (6.2-9.0) | 9.3  (6.1-12.4) |
| Health systems strength | 8.0  (6.9-9.1) | 7.0  (5.8-8.2) | 6.7  (4.2-9.2) |
| Adult COVID-19 coverage | 7.5  (6.3-8.7) | 7.7  (6.3-9.1) | 6.1  (3.2-8.9) |
| TB burden among children | 6.0  (4.9-7.1) | 6.9  (5.6-8.3) | 8.0  (5.0-11.0) |
| Burden of drug resistant-TB | 6.6  (5.4-7.9) | 6.0  (4.5-7.4) | 5.4  (3.1-7.7) |
| Favorable regulatory processes | 6.1  (5.1-7.2) | 6.4  (5.0-7.8) | 6.2  (3.9-8.5) |
| Financial commitment to TB | 5.8  (4.7-6.9) | 6.7  (5.3-8.1) | 6.3  (4.3-8.2) |
| Infant DPT3 coverage | 6.4  (5.3-7.6) | 5.5  (4.3-6.6) | 3.9  (2.7-5.2) |
| Gavi eligibility | 5.1  (4.0-6.1) | 4.5  (3.4-5.6) | 6.8  (4.2-9.4) |
| Short-course TPT introduction | 5.1  (4.1-6.1) | 4.0  (2.8-5.3) | 4.6  (2.1-7.1) |
| Adolescent HPV vaccine introduction | 4.8  (3.9-5.8) | 4.0  (3.0-5.0) | 4.2  (2.4-6.0) |
| HIV-associated TB burden | 3.8  (3.0-4.6) | 3.9  (2.6-5.2) | 3.4  (1.5-5.2) |
| Infant BCG coverage | 3.6  (2.9-4.4) | 3.5  (2.7-4.3) | 3.8  (2.3-5.3) |
| Participation in TB vaccine trials | 3.1  (2.2-3.9) | 3.2  (2.1-4.3) | 3.8  (1.4-6.2) |

DPT3: diphtheria-pertussis- tetanus; TPT: TB preventive therapy; HPV: human papillomavirus; BCG: Bacille Calmette-Guérin.
