# Supplementary material for: Prioritizing countries for TB vaccine readiness research using a global stakeholder-centric approach
Source: PLOS Glob Public Health. 2025 Aug 1;5(8):e0004668. doi: 10.1371/journal.pgph.0004668 (PMC12316289; doi:10.1371/journal.pgph.0004668)
Supplement: S6 Table — (DOCX) [file pgph.0004668.s006.docx]

**S6 Table. Best-Worst Scaling Count Analysis (n=115)**

| Statement | Times Shown | Times Selected Best | Best Count Proportion | Times Selected Worst | Worst Count Proportion | BWS Score* | Ranks |
| --- | --- | --- | --- | --- | --- | --- | --- |
| Overall TB burden | 355 | 170 | 0.48 | 33 | 0.09 | 0.39 | 1 |
| HIV-associated TB burden | 350 | 50 | 0.14 | 123 | 0.35 | -0.21 | 15 |
| TB burden among children | 352 | 90 | 0.26 | 63 | 0.18 | 0.08 | 6 |
| Burden of drug resistant-TB | 353 | 89 | 0.25 | 81 | 0.23 | 0.02 | 9 |
| Burden of TB-related deaths | 351 | 110 | 0.31 | 51 | 0.15 | 0.17 | 3 |
| Infant BCG coverage | 353 | 54 | 0.15 | 103 | 0.29 | -0.14 | 14 |
| Infant DPT3 coverage | 349 | 91 | 0.26 | 80 | 0.23 | 0.03 | 8 |
| Adolescent HPV vaccine introduction | 351 | 65 | 0.19 | 91 | 0.26 | -0.07 | 12 |
| Adult COVID-19 coverage | 351 | 108 | 0.31 | 63 | 0.18 | 0.13 | 5 |
| Health systems strength | 353 | 113 | 0.32 | 56 | 0.16 | 0.16 | 4 |
| Gavi eligibility | 350 | 70 | 0.20 | 88 | 0.25 | -0.05 | 11 |
| Vaccine manufacturing capacity | 349 | 29 | 0.08 | 230 | 0.66 | -0.58 | 17 |
| Favorable regulatory processes | 351 | 94 | 0.27 | 71 | 0.20 | 0.07 | 7 |
| Financial commitment to TB | 353 | 88 | 0.25 | 81 | 0.23 | 0.02 | 10 |
| Short-course TPT introduction | 354 | 65 | 0.18 | 101 | 0.29 | -0.10 | 13 |
| Political commitment to end TB | 350 | 155 | 0.44 | 39 | 0.11 | 0.33 | 2 |

DPT3: diphtheria-pertussis- tetanus; TPT: TB preventive therapy; HPV: human papillomavirus; BCG: Bacille Calmette-Guérin.

*BWS score determined by taking the Best Count Proportion minus the Worst Count Proportion
