# Supplementary material for: Prioritizing countries for TB vaccine readiness research using a global stakeholder-centric approach
Source: PLOS Glob Public Health. 2025 Aug 1;5(8):e0004668. doi: 10.1371/journal.pgph.0004668 (PMC12316289; doi:10.1371/journal.pgph.0004668)
Supplement: S7 Table — (DOCX) [file pgph.0004668.s007.docx]

| **Country Identifier Code** | **Using 16 Criteria Deemed Important** | | **Using all 17 Original Criteria** | | **Change in Ranking** |
| --- | --- | --- | --- | --- | --- |
|  | **Country Prioritization Score** | **Ranking** | **Country Prioritization Score** | **Ranking** |  |
| 18 | 49.5 | 10 | 50.2 | 9 | +1 |
| 5 | 47.5 | 12 | 48.3 | 12 | 0 |
| 3 | 48.4 | 11 | 48.4 | 11 | 0 |
| 19 | 39.4 | 21 | 38.8 | 21 | 0 |
| 20 | 40.5 | 20 | 40.6 | 20 | 0 |
| 7 | 51.0 | 6 | 51.8 | 6 | 0 |
| 12 | 47.1 | 13 | 47.9 | 13 | 0 |
| 16 | 59.1 | 2 | 58.9 | 2 | 0 |
| 21 | 46.8 | 15 | 46.1 | 16 | -1 |
| 22 | 46.9 | 14 | 46.2 | 15 | -1 |
| 4 | 46.8 | 16 | 46.1 | 17 | -1 |
| 11 | 32.1 | 23 | 32.4 | 23 | 0 |
| 6 | 50.1 | 9 | 50.8 | 8 | +1 |
| 9 | 50.3 | 8 | 51.1 | 7 | +1 |
| 23 | 74.8 | 1 | 75.2 | 1 | 0 |
| 13 | 39.1 | 22 | 38.4 | 22 | 0 |
| 2 | 57.2 | 4 | 56.3 | 4 | 0 |
| 14 | 53.3 | 5 | 52.5 | 5 | 0 |
| 15 | 41.7 | 18 | 41.0 | 19 | -1 |
| 10 | 45.9 | 17 | 46.7 | 14 | +3 |
| 1 | 40.9 | 19 | 41.8 | 18 | +1 |
| 17 | 57.2 | 3 | 56.3 | 3 | 0 |
| 8 | 50.5 | 7 | 49.7 | 10 | -3 |

**S7 Table. Sensitivity Analysis Examining the Effect of Including all 17 Criteria on Country Prioritization Scores and Overall Ranking**
